# Fanconi syndrome induced by topical cidofovir in an otherwise healthy young woman

**DOI:** 10.1007/s40620-025-02343-0

**Published:** 2025-06-25

**Authors:** Birgit Korinek, Ursula Thiem, Florian Pfaff, Afschin Soleiman, Bernhard Kirsch

**Affiliations:** 1 Department of Internal Medicine III, Nephrology and Diabetology, Landesklinikum Mistelbach-Gänserndorf, Liechtensteinstrasse 67, 2130 Mistelbach, Austria; 2 Medical Faculty, Johannes Kepler University Linz, Linz, Austria; 3 Department of Internal Medicine I, Cardiology and Intensive Care Medicine, Landesklinikum Mistelbach-Gänserndorf, Mistelbach, Austria; 4 PiZ, Patho Im Zentrum GmbH, St. Pölten, Austria

**Keywords:** Acute kidney injury, Cidofovir, Fanconi syndrome, Topical drug delivery

## The case

A 46-year-old woman presented to the emergency department with a 4-day history of weakness, nausea, and emesis. She had no medical history except for genital condyloma and cervical infection with human papillomavirus (HPV) high-risk subtypes 16 and 18. Despite cervical conization and topical treatment with trichloroacetic acid and imiquimod, infection with HPV 16 persisted. Therefore, she started intravaginal administration of 2% cidofovir gel containing hydroxyethylcellulose as a vehicle before bedtime for five days, followed by a nine-day interval without therapy. Side effects required dose reduction from 5 to 2.5 ml after the first cycle. On the last (6th) cycle, the full dose of cidofovir was resumed, which was completed three days before hospital admission. 

On hospital admission her vital signs were normal. Physical examination showed a female of normal weight, and abdominal examination was notable for diffuse tenderness to palpation. Initial blood tests revealed an elevated serum creatinine of 1.7 mg/dl - estimated glomerular filtration rate (eGFR 32 ml/min/1.73 m^2^) and mild hypokalemia (3.1 mmol/l). Abdominal x-ray suggested constipation and kidney ultrasound was unremarkable. Exsiccosis required intravenous fluid and potassium replacement.

**Fig. 1:**
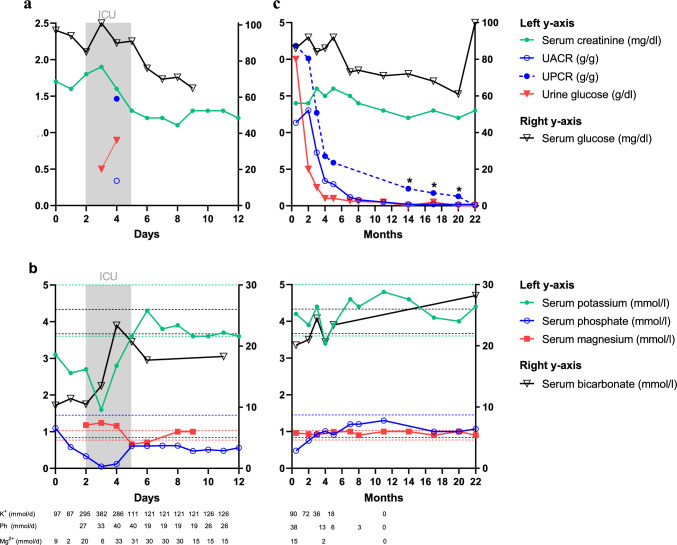
Course of **(a, c)** renal function and **(b, d)** serum electrolytes and daily required electrolyte (potassium, phosphate, magnesium) supply during hospitalization **(a, b)** and outpatient care **(c, d)**. The grey shaded area indicates treatment at the intensive care unit. Dashed lines indicate the reference ranges of the respective parameter. UACR urine albumin-creatinine ratio, UPCR urine protein-creatinine ratio, Asterisks indicate 24-h proteinuria (g/24 h)

After two days, she experienced a prolonged syncope with an asystolic phase of eleven seconds followed by bradycardia (25 bpm) with *U*-waves. Echocardiography showed a hyperdynamic left ventricle indicative of hypovolemia. Blood test revealed metabolic acidosis (pH 7.17, base excess − 14.3 mmol/l), severe hypokalemia (1.6 mmol/l), hypophosphatemia (< 0.10 mmol/l), hypomagnesemia, and hypouricemia (1.0 mg/dl). Urine analysis revealed proteinuria (urine protein to creatinine ratio [UPCR] 1460 mg/g, urine albumin to creatinine ratio [UACR] 229 mg/g) without microhematuria, but with pronounced glucosuria (895 mg/dl) at normal blood glucose levels, and normal HbA1c of 4.7%, indicating proximal tubular injury (Fig. 1). In the intensive care unit, she received continuous, high dose infusions of potassium malate, magnesium sulphate, and glucose-1-phosphate (Fig. 1a, b).

After electrolyte and fluid replacement, her cardiac rhythm stabilized, and her kidney function and general health gradually improved. After discharge, serum electrolytes were stable with oral supplements. As elevated creatinine levels and proteinuria persisted after three months (Fig. 1c, d), she underwent kidney biopsy. Histopathological examination revealed interstitial nephritis with acute and chronic tubular damage (Supplementary Fig. 1) consistent with Fanconi syndrome. Even after almost two years, kidney function did not fully recover. 

### Lesson for the clinical nephrologist

Cidofovir is approved for intravenous treatment of cytomegalovirus retinitis in adults with acquired immunodeficiency syndrome [1]. As it also exerts activity against HPV, topically applied cidofovir is used off-label to treat HPV-associated skin lesions [2]. Intravenous cidofovir is nephrotoxic, selectively injuring proximal tubule cells [3], thereby causing Fanconi syndrome and acute kidney injury (AKI) [1]. Limited data exist concerning systemic toxicity from topical cidofovir.

We report the first case of a kidney-healthy adult presenting with AKI and severe Fanconi syndrome following intravaginal application of cidofovir for HPV infection. To date, only few cases of AKI and proximal tubular damage following topical cidofovir have been published [4–6]. Notably, all of these patients had either pre-existing chronic kidney disease [4–6], and/or had undergone stem cell [4, 6] or solid organ [5] transplantation and thus were being treated with other tubulotoxic drugs such as cyclosporine A, tacrolimus, foscarnet, amikacin or acyclovir.

Notably, in a randomized trial involving 180 women treated with 1% cidofovir gel thrice weekly for vulval intraepithelial neoplasia, the number of patients with proteinuria increased from 4 to 23% after six months [7]. However, the extent of proteinuria, kidney function or serum electrolytes was not specified. In contrast, a phase II trial in 30 patients using 1% cidofovir gel for HPV-associated genital and/or perianal warts reported only local adverse effects like pain, pruritus, and rash [2]. Nevertheless, disease and treatment conditions differed in several aspects in our patient. First, she used a gel with higher cidofovir concentration. Moreover, while in the aforementioned trials cidofovir gel was applied only on external skin, our patient applied it intravaginally without removing residual gel the following morning. Preclinical pharmacokinetic studies suggest that cidofovir absorption varies by application site, with bioavailability being 20-times higher on damaged skin compared with intact skin [8]. To date, bioavailability studies using topical cidofovir on mucous membranes are lacking. Not only is the permeability of mucous membranes higher than that of external skin, but numerous other factors may specifically influence drug uptake from the vaginal route like the large surface area and blood supply for the vaginal epithelium, the volume, composition, and pH of the vaginal fluid or the thickness of the vaginal epithelium, which strongly depends on age and hormonal levels. For other drugs like betablockers, animal studies indeed confirmed that intravaginal administration leads to higher serum concentrations and 36-times higher bioavailability than oral administration [9]. Thus, different drug release and uptake kinetics in the vaginal environment compared with the skin could explain unexpectedly high serum concentrations causing systemic toxicity.

Although serum cidofovir levels were not measured, causality between topical cidofovir therapy and kidney damage is supported by indirect evidence. Firstly, there is a close temporal link between cidofovir therapy and symptom onset and abnormal laboratory findings similar to previous case reports where AKI occurred five to seven days after treatment [4–6]. Secondly, after the first cycle of topical cidofovir, our patient developed nausea and emesis, common side effects of intravenous cidofovir. These symptoms improved after dose reduction, but worsened with resumption of the full dose. Thirdly, cidofovir is known to be tubulotoxic, and tubular cell injury in this patient was confirmed by renal biopsy, with histological findings similar to a previous case of cidofovir-induced renal damage [3]. Importantly, no other factors explaining acute kidney and tubular injury were identified, and prior blood and urine tests provide evidence against pre-existing kidney disease. Using the Naranjo algorithm, a score of 7 indicated a probable adverse drug reaction to topical cidofovir.

In summary, this case illustrates that intravaginally-applied cidofovir may cause serious and potentially life-threatening adverse events even in kidney-healthy adults. It also highlights the need for further pharmacokinetic studies of topical cidofovir. Given the lack of data concerning these issues and the off-label use of cidofovir, we recommend caution when prescribing cidofovir topically and propose vigilant monitoring of serum electrolytes and kidney function by serum creatinine and urine dipstick test. 

## Data availability statement

The datasets generated and analyzed during this case study are available from the corresponding author on reasonable request.

## Supplementary Information

Below is the link to the electronic supplementary material.

Supplementary file1 (DOCX 615 KB)
