# Supplementary material for: Fanconi syndrome induced by topical cidofovir in an otherwise healthy young woman
Source: J Nephrol. 2025 Jun 25;38(5):1501–4. doi: 10.1007/s40620-025-02343-0 (PMC12289827; doi:10.1007/s40620-025-02343-0)
Supplement: Supplementary file 1 — Supplementary file1 (DOCX 615 KB) [file 40620_2025_2343_MOESM1_ESM.docx]

**
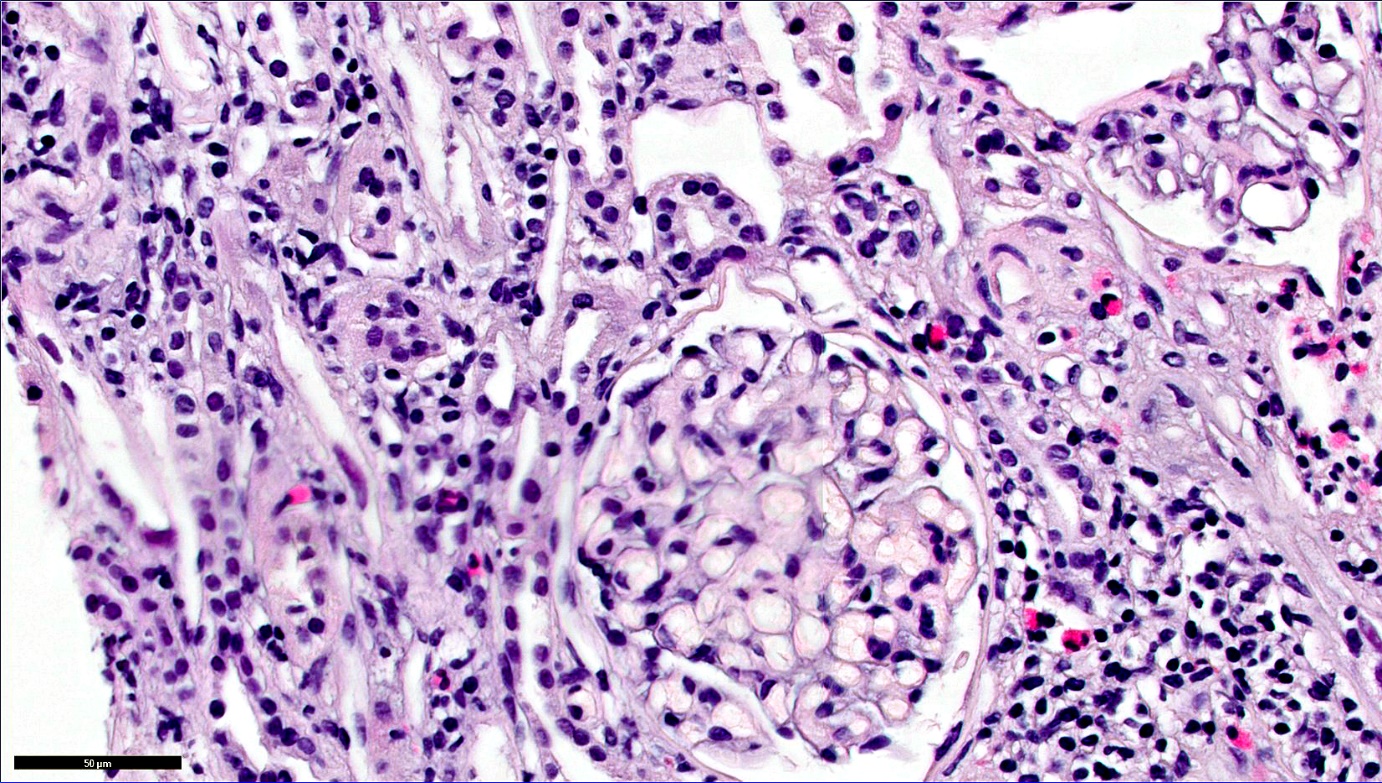
Supplementary Figure 1**

**Supplementary Fig. 1** Hematoxylin and eosin staining of renal biopsy specimens showing mixed inflammatory interstitial infiltrates marked with arrows and acute and chronic tubular damage.
